# Reducing the osteoporosis treatment gap: an interview with UCB's Pascale Richetta

**DOI:** 10.4155/fsoa-2017-0110

**Published:** 2017-11-22

**Authors:** Pascale Richetta

**Affiliations:** 1 UCB Pharma, Brussels, Belgium

**Keywords:** Capture the Fracture, EULAR, fracture risk, fragility fracture, osteoporosis, UCB

## Abstract

Dr. Pascale Richetta is Executive Vice President and Head of the Bone Patient Value Unit at UCB (Brussels, Belgium). She joined UCB in 2016 following over 20 years’ experience in the pharma and biotech industry. After completing her MD at the University of Poitiers (France), she spent time as vice president at Abbvie/Abbott and has also held positions at GlaxoSmithKline, Ipsen and Servier. Through the course of her career she acquired impressive commercial experience managing both traditional pharmaceuticals and complex biologics and her knowledge of markets around the world is key to helping UCB prepare for future product launches.

## Could you tell us a little about your career to date?

I started my professional career as a physician, but my first roles in pharma were in France (where I grew up) at Servier and Ipsen Biotech. I loved working in this space; it meant, I could be hands on in helping develop new medicines and solutions for patients in a scientific setting. This then led to 10 years at GlaxoSmithKline, on both sides of the channel, where I focused on neuroscience, particularly psychiatry and neurology. This was challenging, but very rewarding. In 2007, I moved to Abbott then Abbvie, the speciality care arm of Abbott, as Vice President for Western Europe and Canada. In this role, I worked across a wide range of therapeutic areas, including complex biologics, and I was responsible for overseeing 19 affiliates, totaling more than 3000 staff. I joined UCB a year and a half ago as the head of the Bone Patient Value Unit (bPVU), and my main aim is to take UCB's investigational osteoporosis medicine, which is being co-developed with Amgen, through its development phases and upon regulatory approval ensure we are prepared for its worldwide launch.

## What does your position at UCB involve?

My aim as head of the bPVU is to support the larger mission of UCB; we are very much an innovation-driven global biopharmaceutical company focused on addressing unmet needs and creating value for patients by improving their lives and thereby creating value for society in general. In the bPVU, the goal of creating patient value begins with an integrated team progressing our investigational osteoporosis medicine from Phase II, all the way through to commercialization. The other aspect that we are focused on is working to address the unmet medical need in post-fracture care. We are currently seeing a gap in the treatment and management of osteoporosis, especially in the post-fracture setting, with one out of five patients remaining undiagnosed and untreated [1]. Without adequate management, patients with osteoporosis often face a loss of productivity and independence. In fact, evidence and our experience with patients both show that loss of independence is a concern for osteoporosis patients [2]. The medical, social and personal cost of this disease cannot be overstated, and at UCB, we are making it our priority to reverse this trend.

## UCB recently released the results of a real-world study indicating that the amount of patients receiving osteoporosis treatment after their first fracture in Sweden (6.6%) is much lower than the target (30%). Could you tell us a little more about the study results?

This work is being jointly led by our real-word evidence and medical teams alongside Kristina Åkesson at Skane University Hospital, Lund, in Sweden. We wanted to understand what happened in the real world once a patient experiences their first fragility fracture and whether they receive adequate management and treatment for osteoporosis. Sweden is one of the few countries that has a strong, high-quality national database, so we could review the patient data from the Swedish national registers from 2006 to 2012 and assess the situation. Kristina Åkesson presented this data analysis (from 2006 to 2012) in her recent presentation at European League Against Rheumatism (EULAR) Congress, which showed that only 6.6% of treatment-naive patients were prescribed osteoporosis treatment within 1 year of their first fracture [3].

This rate of non-treatment is a cause for concern, particularly in such a potentially life-altering disease. Given that new Swedish recommendations regarding post-fracture care were published in 2012, the results we see here can act as a ‘time zero’ allowing us to monitor future progress, especially now that fracture liaison services (FLS) are being further developed in Sweden. In addition, Sweden is one of the few countries, and the only one in Europe, that has a bold target to treat 30% of all osteoporosis patients.

We believe that low rates of osteoporosis treatment post-fracture are a wide-scale problem. For example, preliminary figures in the UK [4] show that before the introduction of the FLS into the UK healthcare system, osteoporosis treatment rates were also around 6%.

## Why do you think the figure is only 6%?

At this time, we can only make assumptions about why the figure is so low. If you take a few minutes to consider what happens when a patient suffers from an osteoporotic fracture, it is clear that the system is very fragmented. When someone turns up to their doctor with a clinical fracture they will be referred onto a surgeon or an emergency unit for a plaster cast or splint. In both instances, when the plaster cast or splint is fitted, the problem is then deemed to be ‘fixed’. In this common scenario, osteoporosis is not at the front of the healthcare professional or patient's mind, so the story will then end. There is no obvious link being made between the fracture and the possibility of osteoporosis – the fracture is simply considered in isolation. But this should not be the case. A fracture should be seen as a warning sign that triggers screening for osteoporosis and the initiation of adequate management to prevent further fractures. To overcome this, FLS should work to systematically connect the surgery and/or the emergency unit with the department that deals with osteoporosis care, so that any patient that has a fracture and is, say, above 50–60 years old has a systematic check of their bone mineral density (BMD). Then, if the figures are low enough to justify treatment, it can be initiated. Unfortunately, at present, the patient rarely questions whether they have an underlying condition such as osteoporosis.

## What would you say needs to be done to achieve this ideal process of care?

When you talk to the scientific community, it becomes clear that everyone is enthusiastic for a FLS – a system that really connects all the services. For example, this could be the introduction of a single database that can identify at-risk fracture patients and connect them to a specialist who can determine their bone mineral density and whether they require treatment for osteoporosis. This quick identification and turnaround is critical, considering that when you face one fracture, your risk of a subsequent fracture is highest in the first year following the initial fracture. A FLS can potenitally prevent another fracture, which could impair a patient's quality of life. The name of the game is to connect the dots so there a smooth flow from fix of a fracture, to treatment of the underlying osteoporosis.

## How is UCB planning to help reduce the treatment gap?

One of our teams is actively working to close the treatment gap through improving osteoporosis post-fracture care by working directly with the scientific community to foster the adoption and implementation of post-fracture care models and providing education on the cause of fragility fractures. At this year's EULAR, new guidance was published which included recommendations for the management of patients over 50 with a fragility fracture and prevention of subsequent fracture [5]. These recommendations now cover ten points that aim to ensure that patients have access to the right care. For example, making sure that each clinic that treats fractures has a bone lead assigned who aims to check bone mineral density (BMD) and makes recommendations on appropriate management and treatment as early as possible.

Piet Guesens (Maastricht University Medical Center, Maastricht, The Netherlands) has been leading this effort for EULAR. It is worth mentioning here that the EULAR/EFORT recommendations are not only from specialists in charge of treating osteoporosis, but also from surgeons. It is a really strong collaborative effort and a necessary starting point going forward. The heart of the work now will be to roll-out the recommended changes at a national level and then focus on clinics and hospitals at a more granular level. UCB aims to provide support for this group via our medical liaison staff.

What is more, in our Japanese and USA operations, we have regional care leads that are connecting with healthcare institutions, helping them to assess what is already in place, and how to improve efficiency of the care system.

## How long do you think it will be before these projects are rolled out?

Time is of the essence but we cannot do this overnight. A mixture of support is needed, from improving the efficiency of some FLS already in place, to figuring out how to start a FLS in other sectors. The International Osteoporosis Foundation, for example, has released an international toolkit to help doctors get started with the process [6]. The toolkit gives those wanting to establish a FLS the case for, and the resources to, enable FLS expansion. Similarly, the US National Osteoporosis Foundation is offering a large range of supporting tools on their website. Our aim at UCB is to support the scientific and medical community with these types of initiatives both at a macro- and local-level.

## Finally, taking a broader view of the general osteoporosis field, what are your hopes for the future?

My hope for the future is that the unmet medical need and current care gap in post-fracture management is fully recognized and addressed. I would like to see an increase in osteoporosis screening and diagnosis, to ensure that every high-risk fracture patient has their BMD assessed and managed appropriately to avoid further, debilitating fractures. Osteoporosis should not be a life-altering disease.
